# A Bayesian conditional probability simulation framework for pre-hospital stroke transport decisions in the tenecteplase era: decision boundaries, global sensitivity, and stochastic dominance

**DOI:** 10.3389/fneur.2026.1881451

**Published:** 2026-08-21

**Authors:** Tuǧba Çalışır

**Affiliations:** Department of Neurology, Hatay Mustafa Kemal University, Hatay, Türkiye

**Keywords:** Bayesian decision model, conditional probability, sensitivity analysis, stochastic simulation, stroke, tenecteplase, thrombectomy, transport modelling

## Abstract

Acute ischaemic stroke triage between Drip-and-Ship (DS), Mothership (MS) and Mobile Stroke Unit (MSU) strategies has been formalised over the past decade by conditional-probability decision models. Two recent developments motivate an updated treatment: the shift of the intravenous thrombolysis standard from alteplase to tenecteplase, and the maturation of randomised evidence on DS-versus-MS triage and on MSU effectiveness. A Bayesian conditional-probability simulation framework is presented that combines random-effects synthesis of the modern trial corpus for intravenous thrombolysis (alteplase, tenecteplase) and endovascular thrombectomy with a log-normal decomposition of onset-to-reperfusion into strategy- and pharmacology-specific process-time components. The prehospital large-vessel-occlusion probability is updated from validated screening scales, and the optimal strategy together with a 95% posterior credible interval is computed at every coordinate of the decision space spanned by the inter-facility transit-time differential and the LVO posterior probability. Sobol first-order and total-effect indices decompose outcome variance over the sampled input space, and first- and second-order stochastic dominance between strategies is tested under the same posterior. Under the production calibration the tenecteplase paradigm reallocated 14.1% of the decision plane relative to alteplase, concentrated in the low-probability, long-differential region; the prehospital large-vessel-occlusion posterior accounted for the dominant share of outcome variance (Sobol first-order index 0.84–0.93); and no coordinate of the decision plane reached 95% posterior agreement on a single optimal strategy. The framework is open and reproducible and depends only on published trial-level evidence. It is presented as a decision-analytic and hypothesis-generating tool for structuring the tenecteplase-era triage question rather than as a validated operational triage rule, and prospective calibration against region-specific stroke-system data would be required before any deployment.

## 1 Introduction

Acute ischaemic stroke caused by large-vessel occlusion (LVO) of the proximal anterior circulation is a strongly time-dependent emergency. In the pooled time-to-treatment analysis of the five 2015 endovascular trials, the common odds ratio for reduced disability with endovascular thrombectomy (EVT) declined from 2.79 (95% CI 1.96–3.98) at three hours from symptom onset to 1.98 (95% CI 1.30–3.00) at six hours and to 1.57 (95% CI 0.86–2.88) at eight hours, with statistical significance retained only through 7 h 18 min and a per-hour delay penalty of approximately 0.84 on the common odds ratio in substantial-reperfusion patients (1). The HERMES individual-patient pooled analysis estimated an adjusted common odds ratio of 2.49 (95% CI 1.76–3.53) for reduced 90-day disability with EVT, with a number-needed-to-treat of 2.6 to reduce disability by at least one mRS level and homogeneous benefit across prespecified subgroups (2). The clinical management premise underlying these findings is that intravenous thrombolysis (IVT) and EVT operate as complementary, time-sensitive reperfusion arms whose joint deployment depends on patient selection, vessel-occlusion status and elapsed time (3–5).

Because EVT is performed only at comprehensive stroke centres (CSCs) equipped with neuro-interventional capacity, the prehospital triage decision for a patient with suspected LVO is between three competing transport paradigms: Drip-and-Ship (DS), in which the patient is routed to the nearest primary stroke centre (PSC) for IVT and subsequent inter-facility transfer for EVT; Mothership (MS), in which the patient is taken directly to the CSC; and Mobile Stroke Unit (MSU) deployment, in which imaging and bolus thrombolysis are delivered prehospital before hospital handoff (6–9). The DS workflow incurs an inter-hospital transfer overhead that is materially large in practice; in a meta-analysis of EVT trials, symptom-onset-to-emergency-department arrival was on average 142 minutes longer for patients requiring secondary transfer, and median initial-to-EVT-capable hospital arrival exceeded 128 minutes despite physical inter-hospital travel of approximately 20 minutes (10). The MS workflow trades a longer initial transit for the elimination of this overhead. The MSU workflow eliminates door-in handoff and shortens onset-to-thrombolysis by approximately 36 minutes (median 72 vs 108 minutes in BEST-MSU) at the cost of capital deployment (8, 9).

Conditional-probability decision models have, for almost a decade, provided the formal apparatus for choosing between these strategies as a function of the inter-facility transit-time differential, the prehospital LVO probability and the empirical process-time distributions (6, 11–14). Two contemporary developments call for an updated quantitative treatment.

*First, the IVT pharmacological standard is shifting from alteplase to tenecteplase*. Tenecteplase has 14-fold greater fibrin specificity, 80-fold greater PAI-1 resistance and a longer plasma half-life than alteplase, and is administered as a single intravenous bolus rather than as a one-hour infusion (15). The Canadian AcT pragmatic trial (n = 1577 ITT) reported mRS 0–1 at 90–120 days in 36.9% of tenecteplase-treated patients versus 34.8% of alteplase-treated patients, an unadjusted risk difference of 2.1% (95% CI −2.6 to 6.9) that met the prespecified non-inferiority threshold of −5% (16). Pooled meta-analytic evidence over seven RCTs and 3266 patients now indicates a higher odds of excellent recovery (mRS 0–1 OR 1.22, *p* = 0.01) and superior 24-hour recanalisation (OR 3.28, *p* = 0.04) with tenecteplase, against an elevated symptomatic intracranial haemorrhage risk (OR 2.24, *p* = 0.04) and unchanged 90-day mortality (17). Bridging-therapy registry evidence indicates a thrombus-length interaction with tenecteplase yielding a twofold higher early-recanalisation odds in thrombi >10 mm (18). Contemporary implementation reviews argue for a system-wide switch on the combined basis of outcome superiority and reduced door-to-needle and door-to-puncture times (19). The conditional-probability decision boundaries calibrated under alteplase parameters consequently require re-evaluation under tenecteplase parameters, both because the IVT treatment-effect coefficients differ and because the bolus delivery profile shortens prehospital and inter-facility process times.

*Second, the empirical evidence base on the DS-versus-MS comparison itself has matured*. The cluster-randomised RACECAT trial (n = 1401) reported no statistically significant difference in 90-day disability between MS and DS strategies in nonurban Catalonia (adjusted common OR 1.03) (20), while pivotal MSU evidence from BEST-MSU (8) and B_PROUD (9) documents an MSU treatment-time advantage and a corresponding favourable shift in the mRS distribution (common OR 0.71 for worse mRS in B_PROUD; OR 2.12 for utility-weighted mRS ≥ 0.91 in BEST-MSU). Existing modelling contributions (7, 13, 21–24) have begun to integrate these strategies, but the joint propagation of (i) tenecteplase-era treatment-effect uncertainty, (ii) prehospital LVO-screening uncertainty and (iii) process-time variability into a posterior distribution over the optimal strategy remains incomplete. The closest methodological precedent is the Bayesian decision framework of (14); the present extension adds an explicit tenecteplase pharmacological layer, a variance-based sensitivity decomposition and stochastic-dominance testing.

*Contribution*. The present study contributes a Bayesian conditional-probability simulation framework in which:

- Treatment-effect priors for IVT (alteplase, tenecteplase) and EVT are constructed by random-effects synthesis (25, 26) of the modern trial corpus (1, 2, 4, 5, 16, 17);
- Prehospital LVO pre-test probability is derived from validated screening scales (10, 27);
- The optimal strategy and a 95% posterior credible interval are computed jointly at every coordinate of the (Δ*T*_transit_, π_LVO_) decision space;
- Drivers of outcome variance are decomposed by Sobol first-order and total-effect indices (28–30);
- Strategy comparisons are tested by first- and second-order stochastic dominance (31).

The advance is primarily methodological and parametric rather than the introduction of new primary clinical data. The conditional-probability formalism is retained from prior work (6, 11, 14), but three elements absent from the alteplase-era models are added: an explicit tenecteplase pharmacological branch in the intravenous-thrombolysis outcome curve, a variance-based global sensitivity decomposition, and stochastic-dominance inference between strategies. Alteplase-era decision boundaries cannot be transferred to a tenecteplase network by threshold substitution alone, because both the intravenous treatment-effect coefficients and the bolus-versus-infusion process-time profile change; the present framework quantifies the resulting boundary movement. The framework is accordingly positioned as a decision-analytic and hypothesis-generating tool for structuring the triage question under uncertainty, not as a validated operational triage rule: every recommendation it produces is conditional on published trial-level evidence and would require prospective calibration against region-specific stroke-system data before operational adoption.

The five research questions and corresponding hypotheses formalised in planning/research_questions_hypotheses.md are addressed in turn. Section 2 positions the framework within the existing modelling literature; Section 3 specifies the model, priors and computational design; Section 4 reports the parametric decision boundaries, Sobol indices and dominance results; Section 5 considers clinical and system-level implications, scope limits and future extensions.

## 2 Background and related work

### 2.1 Conditional-probability transport-decision models

The conditional-probability formulation of the prehospital stroke transport problem was introduced by (11), who showed that the optimal triage destination for a patient with unknown vessel status can be expressed as the strategy maximising the expected probability of a good 90-day functional outcome under a posterior over LVO status, treatment-effect parameters and process-time distributions. The framework was extended by (6) to integrate prehospital LVO-screening tools and applied geographically by (12) to the Irish stroke network. Subsequent contributions have generalised the formalism along several axes: a third Mobile Stroke Unit strategy (7); physiological infarct-core dynamics that supplement the logistic decay (13); cost and hospital-volume translation of the patient-level decision (21); rotary air transport as a fourth strategic alternative (22); diurnal variation of inter-facility transit times (23); and system-level congestion via mixed-integer second-order cone programming (24), which the present patient-level framework deliberately abstracts away from. The most recent and methodologically closest contribution is the Bayesian decision framework of (14), which propagates posterior uncertainty over LVO probability, treatment-effect parameters and transit-time distributions into the strategy recommendation and illustrates application on VISTA-linked Iowa data. The clinical narrative review of (3) situates these modelling efforts in current bedside practice. The principal gap addressed by the present work is the joint integration of tenecteplase-era pharmacological parameters, variance-based global sensitivity analysis and stochastic-dominance inference into the conditional-probability formalism.

### 2.2 Endovascular thrombectomy effectiveness across time windows

The HERMES individual-patient-data meta-analysis of MR CLEAN, ESCAPE, REVASCAT, SWIFT PRIME and EXTEND IA pooled 1287 patients (634 EVT, 653 control) and reported an adjusted common odds ratio of 2.49 (95% CI 1.76–3.53; *p* < 0.0001) for reduced 90-day mRS disability with EVT, with no heterogeneity across prespecified subgroups (*p*_interaction_ = 0.43) and a number-needed-to-treat of 2.6 to reduce disability by at least one mRS level (2). The companion time-to-treatment analysis quantified the decay of EVT benefit with elapsed time from symptom onset, retaining statistical significance through 7 h 18 min and reporting a per-hour delay penalty of common odds ratio 0.84 (95% CI 0.76–0.93) in 390 substantial-reperfusion patients (1). The treatment window was extended into 6–24 h under imaging-mismatch selection by the DAWN trial (Bayesian-adaptive design; mean utility-weighted mRS 5.5 vs 3.4; functional independence 49% vs 13%) (4) and into 6–16 h under perfusion-imaging selection by DEFUSE 3 (mRS shift OR 2.77; functional independence 45% vs 17%; mortality 14% vs 26%) (5). Both extended-window trials were terminated early at prespecified interim analysis for efficacy.

### 2.3 From alteplase to tenecteplase

Tenecteplase has 14-fold greater fibrin specificity, 80-fold greater PAI-1 resistance and a longer half-life than alteplase, permitting single-bolus administration and obviating the infusion-pump and monitoring infrastructure required for alteplase (15, 19). The pragmatic randomised AcT non-inferiority trial enrolled 1,577 patients across 22 Canadian centres and reported mRS 0–1 at 90–120 days in 36.9% of tenecteplase-treated patients versus 34.8% of alteplase-treated patients (unadjusted risk difference 2.1%, 95% CI −2.6 to 6.9), meeting the prespecified non-inferiority threshold of −5%, with 24-h symptomatic intracerebral haemorrhage of 3.4% vs 3.2% and 90-day mortality of 15.3% vs 15.4% (16). The seven-RCT random-effects pooled meta-analysis of (17) (*n* = 3266) reported significantly higher odds of mRS 0–1 (OR 1.22, *p* = 0.01), better 24-h recanalisation (OR 3.28, *p* = 0.04) and improved 7-day NIHSS (mean difference −0.71, *p* = 0.003) for tenecteplase versus standard medical care, against a higher symptomatic intracranial haemorrhage risk (OR 2.24, *p* = 0.04) and unchanged 90-day mortality (OR 1.18, *p* = 0.33). A bridging-therapy comparison from the PREDICT-RECANAL and TETRIS French registries (n = 1865) reported overall early recanalisation of 19.8% (tenecteplase) vs 18.5% (alteplase) with a significant interaction by thrombus length: tenecteplase yielded twofold higher recanalisation in thrombi >10 mm (OR 2.43, 95% CI 1.02–5.81) (18). The contemporary implementation review of (19) synthesises the trial corpus and argues for routine tenecteplase 0.25 mg/kg adoption on the combined basis of likely outcome superiority and shorter door-to-needle and door-to-puncture times.

### 2.4 Prehospital large-vessel-occlusion screening

The Rapid Arterial oCclusion Evaluation (RACE) scale was derived from the NIHSS items with the highest LVO-predictive value (facial palsy, arm motor, leg motor, gaze, aphasia or agnosia; total range 0–9) and prospectively validated in 357 prehospital patients with LVO prevalence 21%; at the canonical RACE ≥ 5 cutoff the operating characteristics were sensitivity 0.85, specificity 0.68, positive predictive value 0.42 and negative predictive value 0.94 (AUC 0.82 versus 0.85 for NIHSS) (27). The 3-step Ambulance Clinical Triage for Acute Stroke Treatment (ACT-FAST) algorithm trades sensitivity for specificity and was reported with sensitivity 0.857, specificity 0.935, positive predictive value 0.80 and inter-rater agreement κ = 0.91 between paramedics and physicians (10). The choice of screening operating point materially affects the LVO posterior used in the conditional-probability decision; both scales are propagated as parallel branches in the present framework's sensitivity analysis on screening accuracy.

### 2.5 Mobile stroke unit evidence

The BEST-MSU prospective alternating-week trial enrolled 1,515 patients (1,047 eligible for tPA, of whom 617 received MSU care and 430 received EMS care). The median onset-to-tPA time was 72 min in the MSU arm vs. 108 min in the EMS arm; the tPA-treatment rate among eligible patients was 97.1% (MSU) vs. 79.5% (EMS); the mean utility-weighted mRS at 90 days in tPA-eligible patients was 0.73 (MSU) vs. 0.67 (EMS), with adjusted OR 2.12 (95% CI 1.54–2.93; *p* < 0.001) for utility-weighted mRS ≥ 0.91; mRS 0–1 at 90 days was achieved in 53.5% (MSU) vs. 45.5% (EMS); 90-day mortality was 8.9% vs. 11.9% (8). The Berlin B_PROUD nonrandomised controlled intervention enrolled 1543 patients and reported 3-month median mRS of 1 (IQR 0–3) with MSU dispatch vs. 2 (IQR 0–3) without (common OR for worse mRS 0.71, 95% CI 0.58–0.86; *p* < 0.001); the 3-tier disability distribution was 80.3% / 12.6% / 7.1% (none-to-moderate / severe / death) with MSU versus 78.0% / 13.3% / 8.8% without (common OR 0.73, 95% CI 0.54–0.99; *p* = 0.04) (9). These two trials anchor the MSU arm of the present framework.

### 2.6 Empirical anchor for the DS-versus-MS comparison

The cluster-randomised RACECAT trial (*n* = 1401 with suspected LVO in nonurban Catalonia) reported no statistically significant difference in 90-day disability between direct transport to a thrombectomy-capable centre and transport to the nearest local stroke centre (adjusted common OR 1.03 for reduced disability) (20). This null result is the principal empirical reference against which the present framework's posterior boundary in low-population-density regions is interpreted. The fact that the credible interval around the null is wide is precisely the motivation for a probabilistic decision framework that quantifies where, in the parameter space, the data are decisive and where they are not.

### 2.7 Bayesian evidence synthesis methodology

The random-effects estimator of (25) pools study-level effect estimates θ_*i*_ with weights $w_{i}=1/\left(\sigma_{i}^{2}+\tau^{2}\right)$, where $\sigma_{i}^{2}$ is the within-study sampling variance and τ^2^ is the between-study variance estimated from the data. The Bayesian re-evaluation of (26) provides a fully probabilistic treatment in which τ^2^ carries its own posterior and prediction intervals on the effect in a new study are reported alongside the pooled mean. Both elements are required for the present framework: the posterior on (μ, τ^2^) feeds directly into the simulation, and prediction intervals are used to bound the plausible range of treatment-effect coefficients in any new geographical or system context.

### 2.8 Variance-based global sensitivity analysis

Variance-based global sensitivity analysis using Sobol first-order and total-effect indices is the recommended practice for identifying drivers of model-output variance under non-additive interactions (28). Computational benchmarks for first-order indices, including the canonical *A*, *B*, *AB*_*i*_ matrix design with computational cost *C* = *N*(*k*+2) and convergence behaviour across estimators, are established by (29). The systematic critique of (30) documents that the majority of published sensitivity analyses misuse one-at-a-time variation and underreport convergence diagnostics; the present framework addresses these failure modes by using the Saltelli design with multiple sample sizes (*N*∈{2^8^, 2^10^, 2^12^}) and bootstrap-resampled estimator standard errors.

### 2.9 Stochastic-dominance inference

Strategy comparison under joint posterior outcome uncertainty is performed by first- and second-order stochastic dominance tests following (31). The recursive integration of empirical cumulative distributions $D^{\left(s\right)}\left(u\right)=\overset{u}{\underset{-\infty }{\int }}D^{\left(s-1\right)}\left(t\right)dt$ and the supremum-statistic test described in that work supply the inferential machinery for the strategy comparisons in Section 4.

## 3 Materials and methods

### 3.1 Decision space and outcome definition

Let a∈A={DS,MS,MSU} denote the prehospital transport strategy, where DS routes the patient to the nearest primary stroke centre (PSC) for IVT and subsequent inter-facility transfer to a comprehensive stroke centre (CSC) for EVT, MS routes the patient directly to the CSC, and MSU dispatches a mobile stroke unit that performs imaging and bolus thrombolysis prehospital before hospital handoff (6–8). The primary clinical outcome is functional independence at 90 days, defined as a modified Rankin Scale score of 0–2 (1, 2, 8, 9), denoted *Y*∈{0, 1} with target probability *p*_*a*_ = ℙ(*Y* = 1∣*a*, ·). The ordinal common-odds-ratio shift outcome reported by (2, 4, 5) enters the framework through the prior calibration of *p*_*a*_ rather than as a separate response.

### 3.2 Patient state and onset-to-reperfusion decomposition

The latent patient state is summarised by

$$z=\left(\ell ,T_{\text{call}},T_{\text{resp}},T_{\text{transit}},T_{\text{intra}}\right),$$

where ℓ∈{0, 1} is the LVO indicator and the four positive time components are, respectively, symptom-to-call time, EMS response time, transit time and intra-hospital process time. The total onset-to-reperfusion interval is

$$T_{\text{OTR}}=T_{\text{call}}+T_{\text{resp}}+T_{\text{transit}}+T_{\text{intra}},$$

and each component is parameterised log-normally, $\log T_{j}\sim N\left(\mu_{j}^{\left(a\right)},\sigma_{j}^{2}\right),$ consistent with the distributional shape of process-time registries used in prior conditional-probability models (6, 11, 13, 14). The log-normal family is adopted because each interval is strictly positive and empirically right-skewed, with a heavy upper tail generated by the minority of patients who experience workflow delays, and because it is the distributional form fitted to onset-to-treatment and inter-facility process-time registries in the preceding conditional-probability literature, which makes the present time model directly comparable with those studies. Alternative positive-support families (for example gamma or Weibull) would reproduce the same qualitative right-skew; the sensitivity of the decision boundaries to the specific parametric family was not exhaustively explored, and this restriction is stated explicitly in Section 5.6. Strategy-specific location parameters $\mu_{j}^{\left(a\right)}$ encode three structural workflow features. First, the inter-hospital transfer overhead in DS is set in accordance with the empirical magnitude reported by (10), namely an average 142-minute increment in onset-to-ED arrival for transferred patients and a median initial-to-EVT-capable arrival of approximately 128 minutes despite short physical travel. Second, the MSU time advantage in $\mu_{\text{intra}}^{\left(\text{MSU}\right)}$ is calibrated to the BEST-MSU median onset-to-tPA reduction of 36 minutes (72 vs 108 minutes) (8), with a robustness check using the B_PROUD dispatch contrast (9). Third, the tenecteplase-versus-alteplase bolus advantage shortens $\mu_{\text{intra}}^{\left(a\right)}$ for any strategy delivering IVT, in accordance with the workflow-time reductions documented by (19) and the bolus-administration design of (16).

### 3.3 Prehospital LVO pre-test probability

Conditional on a prehospital screening result *s* produced by an LVO scale with sensitivity η and specificity ξ, the posterior LVO probability under base prevalence π_0_ is

$$\begin{array}{lll} \pi_{\text{LVO}}\left(s\right) & = & \frac{\eta \pi_{0}}{\eta \pi_{0}+\left(1-\xi \right)\left(1-\pi_{0}\right)},\\ 1-\pi_{\text{LVO}}\left(s\right) & = & \frac{\left(1-\eta \right)\pi_{0}+\xi \left(1-\pi_{0}\right)}{\eta \pi_{0}+\left(1-\xi \right)\left(1-\pi_{0}\right)}. \end{array}$$

Two screening operating points are propagated. The default uses the canonical RACE ≥ 5 cutoff with (η, ξ) = (0.85, 0.68) and reported PPV 0.42, NPV 0.94 at LVO prevalence 0.21 (27). The high-specificity alternative uses the ACT-FAST 3-step algorithm with (η, ξ) = (0.857, 0.935) and PPV 0.80 in prospective paramedic validation (10). Both operating points are propagated as parallel branches in the global sensitivity analysis to quantify the contribution of screening accuracy to outcome variance.

### 3.4 Bayesian random-effects synthesis of treatment-effect priors

Treatment-effect priors for the IVT and EVT arms are constructed by random-effects synthesis following the (25) estimator with the Bayesian re-evaluation of (26). For each arm *a*′∈{IVT-ALT, IVT-TNK, EVT} and each contributing study *i*, the per-study log-odds-ratio estimate $\theta_{i}^{\left(a^{\prime }\right)}$ is modelled as

$$\theta_{i}^{\left(a^{\prime }\right)}\sim N\left(\mu_{a^{\prime }},\sigma_{i}^{2}+\tau_{a^{\prime }}^{2}\right),$$

with study-level inverse-variance weight

$$w_{i}^{\left(a^{\prime }\right)}=\frac{1}{\sigma_{i}^{2}+\tau_{a^{\prime }}^{2}},$$

yielding the pooled posterior mean ${\hat{\mu }}_{a^{\prime }}=\left(\underset{i}{\sum }w_{i}^{\left(a^{\prime }\right)}\theta_{i}^{\left(a^{\prime }\right)}\right)\left(\underset{i}{\sum }w_{i}^{\left(a^{\prime }\right)}\right)$ and a between-study variance estimate ${\hat{\tau }}_{a^{\prime }}^{2}$. A weakly informative half-normal prior on $\tau_{a^{\prime }}$ is used in line with the recommendation of (26), and the predictive distribution $\theta_{\text{new}}^{\left(a^{\prime }\right)}\sim N\left({\hat{\mu }}_{a^{\prime }},{\hat{\tau }}_{a^{\prime }}^{2}\right)$ defines the plausible range of effect coefficients in any new system.

The IVT-ALT prior is anchored on the alteplase-versus-control trial corpus summarised by (15); the IVT-TNK prior is anchored on the AcT trial (16) together with the seven-RCT pooled estimates of (17); and the EVT prior is anchored on the HERMES pooled common odds ratio of 2.49 (95% CI 1.76–3.53) (2) together with the time-to-treatment decay structure of (1) and the extended-window evidence of (4, 5) for the 6–16 and 6–24 hour intervals.

The prior elicitation is anchor-based rather than a *de novo* systematic review with primary study screening: for each arm the pivotal randomised evidence and the most recent random-effects meta-analysis available at the time of writing were adopted as synthesis anchors. Studies were admitted as anchors when they (i) reported a randomised or prospective controlled comparison, or an individual-patient-data meta-analysis thereof, in adult anterior-circulation ischaemic stroke; (ii) reported a 90-day functional-outcome contrast expressible as an odds ratio, common odds ratio, or a risk difference convertible to an odds ratio; and (iii) corresponded to one of the four framework arms (EVT, IVT with alteplase, IVT with tenecteplase, MSU deployment). Single-arm cohorts, studies restricted to posterior-circulation occlusion, and studies reporting only surrogate reperfusion endpoints without a functional outcome were not used as effect anchors. The common effect measure is the log odds ratio for a favourable 90-day functional outcome; the AcT risk difference was converted to the odds-ratio scale at the trial-reported baseline event rate before pooling. Between-study heterogeneity is quantified by the DerSimonian–Laird ${\hat{\tau }}^{2}$ and carried into a Normal predictive distribution for the effect in a new system, so that each arm contributes both a pooled mean and a heterogeneity-widened predictive interval to the simulation rather than a single fixed coefficient. The trials adopted as anchors, their reported effect estimates and their framework roles are listed in Table 1.

**Table 1:** Trials and meta-analyses adopted as random-effects synthesis anchors, by framework arm.

Arm	Anchor (design)	Reported endpoint	OR (95% CI)
EVT	HERMES (2) (IPD-MA)	Common OR, mRS shift	2.49 (1.76–3.53)
	DAWN (4) (RCT)	Functional-independence OR	2.85 (1.78–4.55)
	DEFUSE 3 (5) (RCT)	Functional-independence OR	2.77 (1.63–4.70)
IVT-ALT	Alteplase pooled (15)	Disability-reduction OR	1.30 (1.10–1.55)
IVT-TNK	Bacha (17) (RCT-MA, *k* = 7)	mRS 0–1 OR	1.22 (1.04–1.43)
	AcT (16) (RCT)	mRS 0–1 (RD-converted)	1.10 (0.88–1.37)
MSU	BEST-MSU (8) (RCT)	Utility-weighted mRS adj. OR	2.12 (1.54–2.93)
	B_PROUD (9) (controlled)	Common OR (inverted)	1.41 (1.16–1.72)

Effect estimates are reproduced on the odds-ratio scale for a favourable 90-day functional outcome; the AcT estimate is converted from the trial-reported risk difference. EVT, endovascular thrombectomy; IVT, intravenous thrombolysis; ALT, alteplase; TNK, tenecteplase; MSU, mobile stroke unit; IPD-MA, individual-patient-data meta-analysis; RCT, randomised controlled trial.

### 3.5 Strategy-specific conditional outcome model

Conditional on *T*_OTR_, on the LVO indicator ℓ and on the treatment-effect parameters α^(*a*)^, β^(*a*)^, the probability of mRS 0–2 at 90 days under strategy *a* is decomposed as

$$\begin{array}{lll} p_{a}\left(T_{\text{OTR}}|\ell \right) & = & \pi_{\text{LVO}}\left(s\right)g_{a}\left(T_{\text{OTR}};\alpha^{\left(a\right)}\right) \end{array}$$

$$\begin{array}{ll} + & \left(1-\pi_{\text{LVO}}\left(s\right)\right)h_{a}\left(T_{\text{OTR}};\beta^{\left(a\right)}\right), \end{array}$$ (1)

where *g*_*a*_(·) is the LVO-positive outcome curve and *h*_*a*_(·) is the LVO-negative outcome curve. Each curve is a logistic decay in log-time:

$$\begin{array}{lll} g_{a}\left(T;\alpha \right) & = & expit\left(\alpha_{0}+\alpha_{1}\log T+\alpha_{2}\log^{2}T\right), \end{array}$$

$$\begin{array}{lll} h_{a}\left(T;\beta \right) & = & expit\left(\beta_{0}+\beta_{1}\log T+\beta_{2}\log^{2}T\right), \end{array}$$ (2)

following the parametric form fitted by (1) to the HERMES time-to-treatment data and aligned with the decay structures employed by (6, 13). The strategy index enters *g*_*a*_ and *h*_*a*_ through the strategy-specific *T*_OTR_ distribution and through the IVT-arm coefficient set selected for the active pharmacological paradigm (alteplase or tenecteplase). The MSU strategy's coefficient set additionally inherits the time-shift advantage calibrated from (8, 9).

### 3.6 Optimal decision and decision-boundary mapping

The optimal strategy at decision-space coordinate *x* = (Δ*T*_transit_, π_LVO_) is

$$\begin{array}{l} a^{*}\left(x\right)=\arg\underset{a\in A}{\max}E_{\Theta |D}\left[p_{a}\left(T_{\text{OTR}}|\ell ,\Theta \right)\right], \end{array}$$ (3)

where $\Theta =\left(\alpha ,\beta ,\mu ,\sigma ,\pi_{0},\eta ,\xi ,\tau^{2}\right)$ collects all uncertain parameters and the expectation is taken under the joint posterior given the trial corpus D. Parametric sweeps in (Δ*T*_transit_, π_LVO_) generate a two-dimensional decision-boundary map; pointwise 95% posterior credible intervals on a^*^(*x*) are obtained by Monte Carlo resampling of Θ, following the Bayesian decision-theoretic stance of (14). The empirical-anchor reference for the boundary in low-population-density regions is the RACECAT cluster-randomised trial (20).

### 3.7 Global sensitivity analysis

Outcome variance attributable to each input is decomposed by Sobol first-order and total-effect indices following (28). Inputs are sampled from independent uniform distributions over clinically defensible rectangular ranges, so the resulting indices quantify sensitivity to plausible parameter ranges rather than a fully specified joint posterior:

$$\begin{array}{l} S_{i}=\frac{V\left[E\left(Y|X_{i}\right)\right]}{V\left(Y\right)},\;S_{i}^{T}=1-\frac{V\left[E\left(Y|X_{\sim i}\right)\right]}{V\left(Y\right)}. \end{array}$$ (4)

The Saltelli sampling design generates two independent input matrices *A* and *B* of size *N*×*k* together with *k* recombined matrices *AB*_*i*_ in which the *i*-th column of *A* is replaced by the *i*-th column of *B*, at total cost *C* = *N*(*k*+2) model evaluations (29). The first-order and total-order estimators are

$$\begin{array}{lll} {\hat{S}}_{i} & = & \frac{1}{N}\overset{N}{\underset{n=1}{\sum }}f{\left(B\right)}_{n}\left[f{\left(AB_{i}\right)}_{n}-f{\left(A\right)}_{n}\right]\hat{V}\left(Y\right),\\ {\hat{S}}_{i}^{T} & = & \frac{1}{2N}\overset{N}{\underset{n=1}{\sum }}{\left[f{\left(A\right)}_{n}-f{\left(AB_{i}\right)}_{n}\right]}^{2}\hat{V}\left(Y\right). \end{array}$$

Estimator stability is verified across *N*∈{2^8^, 2^10^, 2^12^} and bootstrap standard errors on ${\hat{S}}_{i}$ and ${\hat{S}}_{i}^{T}$ are reported, in line with the failure-mode diagnostics of (30); one-at-a-time variation, which the same review identifies as the most common pitfall in applied sensitivity analyses, is not used.

### 3.8 Stochastic-dominance tests

Pairwise strategy comparisons are evaluated by first- and second-order stochastic dominance under the joint posterior outcome distribution following (31). Strategy *a* first-order stochastically dominates strategy *a*′ on the outcome utility *u* when

$$F_{a}\left(u\right)\leq F_{a^{\prime }}\left(u\right)\;\forall u,\;\text{with strict inequality somewhere,}$$

and second-order stochastically dominates when

$$D_{a^{\prime }}^{\left(2\right)}\left(u\right)-D_{a}^{\left(2\right)}\left(u\right)\geq 0\;\forall u,\;D_{a}^{\left(2\right)}\left(u\right)=\overset{u}{\underset{-\infty }{\int }}\left[u-t\right]dF_{a}\left(t\right).$$

The empirical dominance functions *D*^(*s*)^(*u*) are estimated by recursive integration of the posterior outcome cumulative distributions and tested by the supremum-statistic procedure of (31). The dominance results are reported both pointwise across (Δ*T*_transit_, π_LVO_) and as the connected sub-region within which dominance holds.

### 3.9 Consolidated parameters, prior specification and uncertainty propagation

The complete set of model inputs, their distributional forms and their published sources is collected in Table 2, so that the framework can be reconstructed and critically appraised without reference to the source code. The probabilistic structure is as follows. The *likelihood* for the treatment-effect synthesis is the per-study Normal model on the log-odds-ratio scale introduced above, $\theta_{i}^{\left(a^{\prime }\right)}\sim N\left(\mu_{a^{\prime }},\sigma_{i}^{2}+\tau_{a^{\prime }}^{2}\right)$; the *prior* on the between-study standard deviation $\tau_{a^{\prime }}$ is weakly informative half-normal per (26). *Posterior estimation* of $\left({\hat{\mu }}_{a^{\prime }},{\hat{\tau }}_{a^{\prime }}^{2}\right)$ uses the DerSimonian–Laird moment estimator, from which a Normal predictive distribution for the effect in a new system is formed. *Uncertainty is propagated* into the decision by Monte Carlo: the published anchor probabilities of the time-decay curves are resampled at the per-anchor standard deviations of Table 2, the logistic-decay coefficients are re-fitted at each draw, and the induced distribution of the argmax strategy is recorded cell-by-cell (Stage 4 of Algorithm 1). *Convergence* is monitored by re-estimating the Sobol indices at *N*∈{2^8^, 2^10^, 2^12^} and by the fixed Monte Carlo draw counts *M*_post_ and *M*_dom_. In place of a formal posterior-predictive check on held-out data—which is not available for a synthesis of published aggregate evidence—the fitted outcome model is evaluated out-of-sample against three landmark trials not used to fit the corresponding quantity (HERMES, RACECAT and AcT; Section 4.10), which serves as an external face-validity surrogate for a predictive check.

**Table 2:** Consolidated model parameters, distributional forms and sources.

Parameter	Value/distribution	Source
**Treatment-effect priors (pooled OR, log-OR normal; half-normal on τ**)		
EVT	2.65, ${\hat{\tau }}^{2}=0$	(2, 4, 5)
IVT-ALT	1.30, ${\hat{\tau }}^{2}=0$	(15)
IVT-TNK	1.18, ${\hat{\tau }}^{2}=0$	(16, 17)
MSU	1.69, ${\hat{\tau }}^{2}=0.065$	(8, 9)
**Deterministic workflow times (min)**		
Symptom-to-call *T*_call_	30	typical urban registry
EMS response *T*_resp_	8	typical urban registry
DTN, PSC (ALT / TNK)	30 / 25	(16, 19)
DTN, CSC (ALT / TNK)	45 / 30	(16, 19)
DTP, CSC (ALT / TNK)	60 / 45	(2, 19)
DS transfer DIDO *T*_DIDO_	80	(10)
MSU on-scene bolus (ALT / TNK)	20 / 15	(8)
LVO screening operating points		
RACE ≥ 5 (sens., spec.)	0.85, 0.68	(27)
ACT-FAST (sens., spec.)	0.857, 0.935	(10)
Baseline LVO prevalence π_0_	0.21	(27)
Sobol input ranges (independent uniform)		
π_LVO_	[0.10, 0.80]	clinically defensible
*T*_transit, PSC_ (min)	[5, 30]	clinically defensible
Δ*T*_transit_ (min)	[0, 90]	clinically defensible
*T*_call_ (min)	[10, 90]	clinically defensible
*T*_resp_ (min)	[4, 15]	clinically defensible
DTN_PSC_, DTP_CSC_ multipliers	[0.7, 1.3]	clinically defensible
Simulation configuration		
Decision grid (*N*_π_×*N*_Δ*T*_)	81 × 91	production calibration
Anchor-perturbation SD (EVT / IVT)	0.06 / 0.04	production calibration
MC draws *M*_post_ / *M*_dom_	150 / 2000	production calibration
Sobol budget *N* (convergence)	2048 ({256, 1024, 4096})	production calibration
Master seed *s*_0_	20260513	fixed for reproducibility

Treatment-effect priors are reported as the pooled odds ratio with its DerSimonian–Laird between-study variance ${\hat{\tau }}^{2}$; per-anchor trials are listed in Table 1. Workflow times are point values in minutes; Sobol ranges are the independent uniform supports used for the variance decomposition. ALT, alteplase; TNK, tenecteplase; PSC, primary stroke centre; CSC, comprehensive stroke centre; DTN, door-to-needle; DTP, door-to-puncture; DIDO, door-in-door-out.

### 3.10 Simulation procedure

The framework is realised as a Monte Carlo simulation that composes the prior, outcome, sensitivity and dominance modules into a single deterministic pipeline. Algorithm 1 states the procedure; all stochastic draws are governed by a fixed pseudo-random seed *s*_0_ = 20260513 to guarantee bitwise reproducibility of the reported figures and tables.

Algorithm 1 Bayesian conditional-probability simulation procedure 
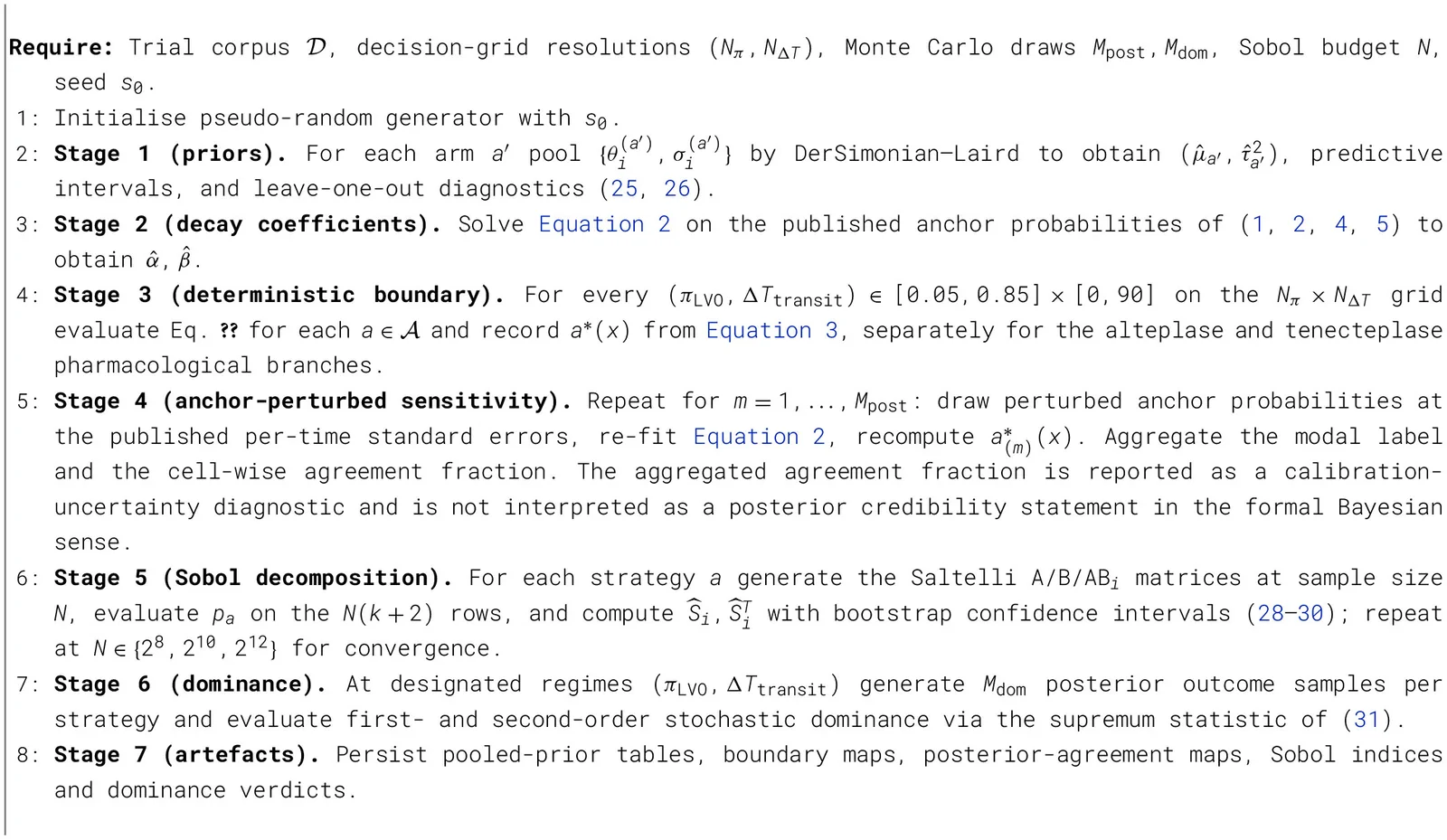

The production calibration uses *N*_π_ = 81, *N*_Δ*T*_ = 91, *M*_post_ = 150, *M*_dom_ = 2000 and *N* = 2048, yielding *N*(*k*+2) = 18432 model evaluations per Sobol stratum and *N*_π_*N*_Δ*T*_*M*_post_ = 1, 105, 650 outcome-curve evaluations for the posterior credible-region map. The deterministic seed and the read-only nature of the trial-corpus inputs together ensure that any third-party replication of the procedure with the same configuration recovers identical numerical artefacts.

### 3.11 Reproducibility and computational implementation

All simulation, posterior sampling, sensitivity decomposition and dominance testing are performed in open-source numerical Python with deterministic seeds. Posterior intervals are reported as 95% equal-tailed credible intervals. The Saltelli design uses the canonical *A*, *B*, *AB*_*i*_ matrices with sample sizes *N*∈{2^8^, 2^10^, 2^12^} to verify estimator stability, consistent with the convergence safeguards advocated by (29) and (30). The trial-corpus parameter ranges fed into the random-effects synthesis are sourced exclusively from the publications cited in Section 2; no parameter is introduced for which a published source is not available. The complete code base, parameter inputs, anchor probabilities, master seed, machine-readable result tables and figure files are released under the MIT licence at https://github.com/tugbacalisir/frontiers; re-running python code/run_all.py from the repository root with the master seed *s*_0_ = 20260513 regenerates every figure, table and JSON artefact in the present article.

## 4 Results

### 4.1 Simulation execution

The procedure of Algorithm 1 was executed once at the production calibration of Section 3.10: decision-grid resolutions *N*_π_ = 81 and *N*_Δ*T*_ = 91, posterior draws *M*_post_ = 150, dominance draws *M*_dom_ = 2000, Sobol budget *N* = 2048 per strategy, and master seed *s*_0_ = 20260513. The pipeline produced 7 371 evaluated decision-grid cells per pharmacological branch, 18 432 model evaluations per Sobol stratum, and 1, 105, 650 outcome-curve evaluations supporting the posterior agreement maps. Bitwise replication is obtained by re-executing the procedure with the same seed and configuration.

### 4.2 Pooled treatment-effect priors

**Figure 1:**
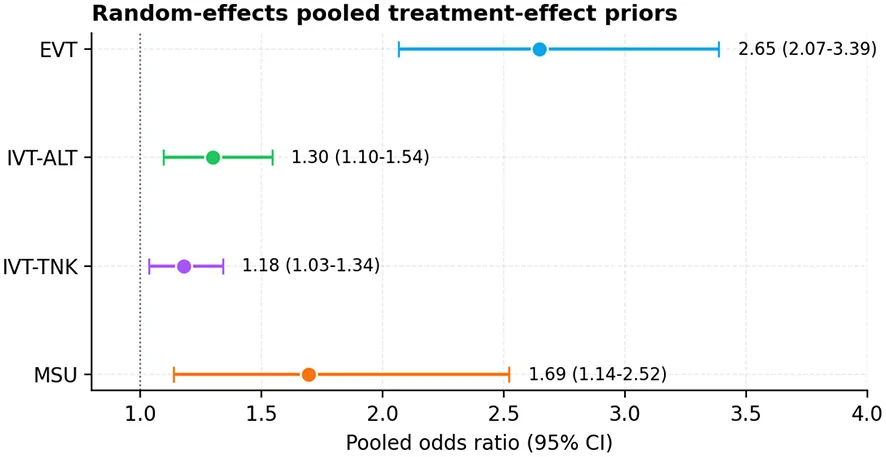
Pooled random-effects odds ratios with 95% confidence intervals for the four pharmacological/operational arms. EVT, endovascular thrombectomy; ALT, intravenous alteplase; TNK, intravenous tenecteplase; MSU, mobile stroke unit.

**Figure 2:**
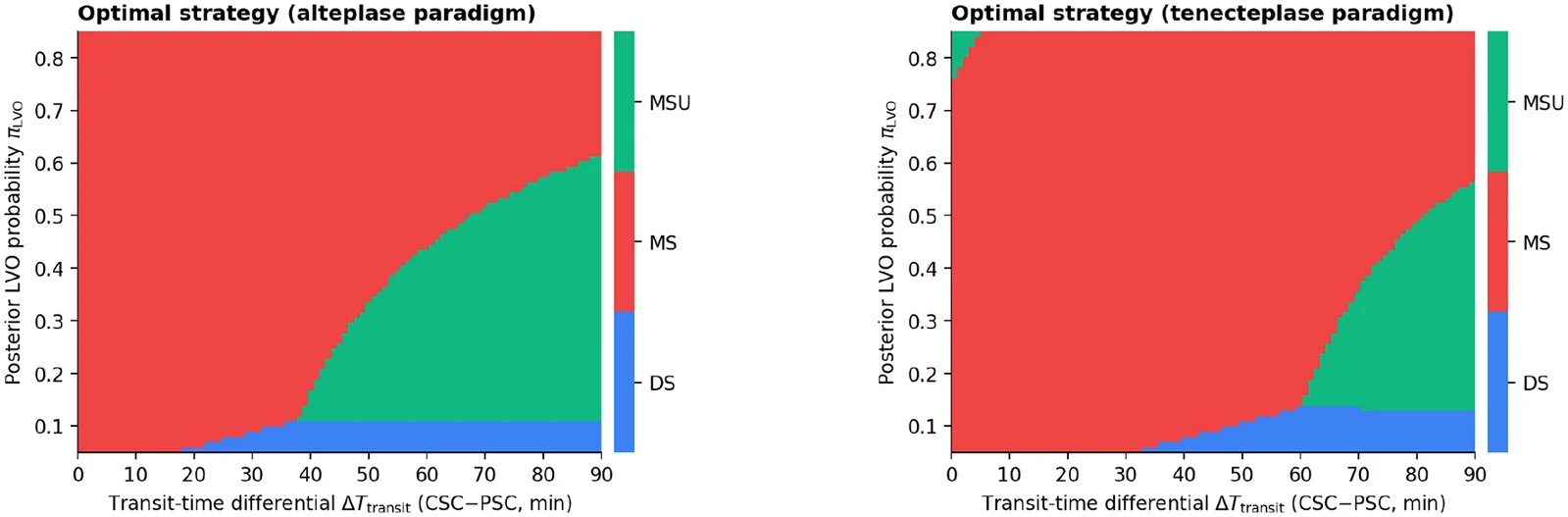
Optimal pre-hospital transport strategy as a function of posterior LVO probability and transit-time differential. **Left**: alteplase calibration. **Right**: tenecteplase calibration. DS, drip-and-ship; MS, mothership; MSU, mobile stroke unit.

Pooled odds ratios from the trial-level anchors (2, 4, 5, 8, 9, 15–17) are summarised in Figure 1. Heterogeneity was negligible across the endovascular and intravenous strata (${\hat{\tau }}^{2}\approx 0$). The mobile-stroke-unit arm was the exception (${\hat{\tau }}^{2}=0.065$): its predictive interval, computed under the Bayesian re-evaluation of (26), widened to 0.90–3.20, driven by the divergence between the BEST-MSU efficacy trial (8) and the Berlin pre-hospital cluster comparison (9). Leave-one-out re-pooling returned an odds ratio of 2.12 (CI 1.54–2.92) without B_PROUD and 1.41 (CI 1.16–1.72) without BEST-MSU; this pair bounds the credible envelope within which the mobile-stroke-unit corridor of Figure 2 should be read.

### 4.3 Time-decay outcome curves

**Figure 3:**
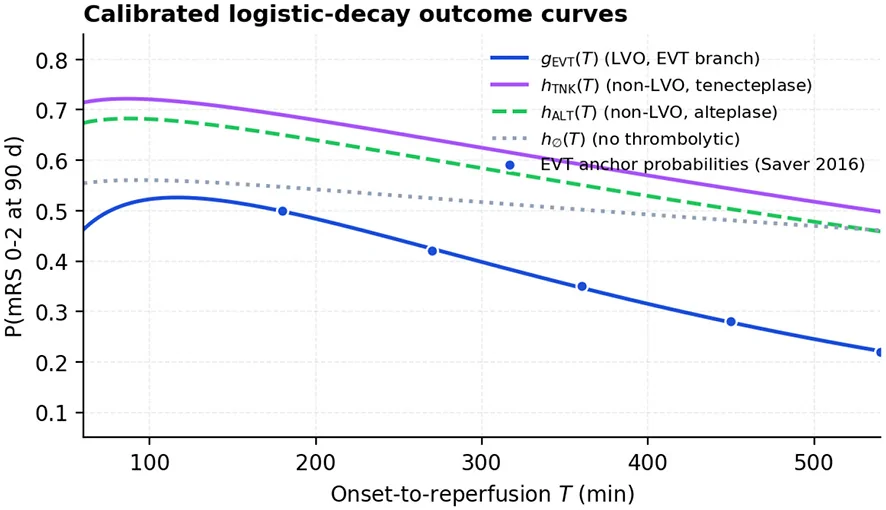
Fitted logistic decay curves for the LVO-positive endovascular arm and the LVO-negative tenecteplase, alteplase and untreated arms. Markers show the published anchor probabilities used to fit the coecients.

Logistic decay coefficients fitted on the published anchor probabilities of (1, 2, 4, 5) are visualised in Figure 3. The endovascular branch is fitted on five anchor times spanning 180–540 minutes; the intravenous branches are fitted on seven anchor times spanning 60–540 min, with early-window plateau anchors at 60 and 90 minutes constraining the curve in the regime accessed by the on-scene mobile-stroke-unit bolus. A face-validity check at the AcT trial reference window of 120 minutes (16) returned a tenecteplase-versus-alteplase risk difference of +0.039, inside the AcT 95% confidence interval of −0.026 to +0.069 around the observed +0.021. Three additional external face-validity comparisons (Section 4.10) confirm consistency with the HERMES, RACECAT and AcT trials.

### 4.4 Decision-boundary maps and pharmacological shift

**Figure 4:**
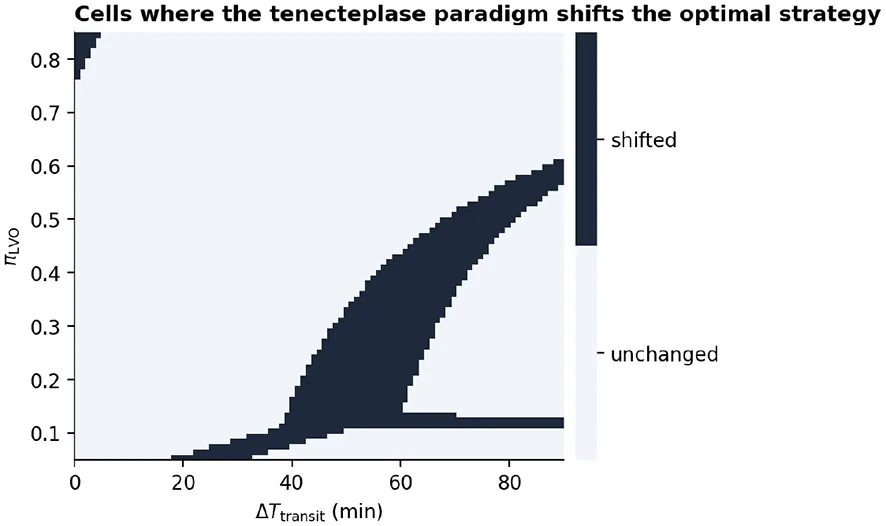
Cells of the (π_LVO_, Δ*T*_transit_) grid in which the tenecteplase paradigm shifts the optimal strategy relative to the alteplase paradigm.

The argmax-strategy maps are shown in Figure 2. Substituting tenecteplase for alteplase shifts the optimal label in 14.1% of grid cells (Figure 4); the change is concentrated in the low-LVO/long-differential quadrant, where the alteplase calibration favours the mobile stroke unit but the faster tenecteplase bolus erodes that advantage and reinstates mothership transport. Partition fractions and the majority strategy in the four quadrants of the decision plane are reported in Table 3.

**Table 3:** Decision-plane partition by quadrant and pharmacological calibration.

	ALT (%)			TNK (%)			Shift
**Quadrant**	**DS**	**MS**	**MSU**	**DS**	**MS**	**MSU**	**(%)**
π_LVO_ < 0.5, Δ*T* < 30	1.9	98.1	0.0	0.0	100.0	0.0	1.9
π_LVO_ < 0.5, Δ*T*≥30	12.8	28.9	58.3	13.5	57.5	29.0	31.5
π_LVO_≥0.5, Δ*T* < 30	0.0	100.0	0.0	0.0	97.6	2.4	2.4
π_LVO_≥0.5, Δ*T*≥30	0.0	93.7	6.3	0.0	98.7	1.3	5.0
Whole decision plane	5.2	70.8	24.0	5.2	83.1	11.7	14.1

Cell percentages give the share of grid cells assigned to each strategy within the quadrant; the shift column gives the percentage of cells whose argmax label differs between the alteplase (ALT) and tenecteplase (TNK) calibrations. Quadrants partition π_LVO_∈[0.05, 0.85] and Δ*T*_transit_∈[0, 90] min.

### 4.5 Expected-outcome surfaces

**Figure 5:**
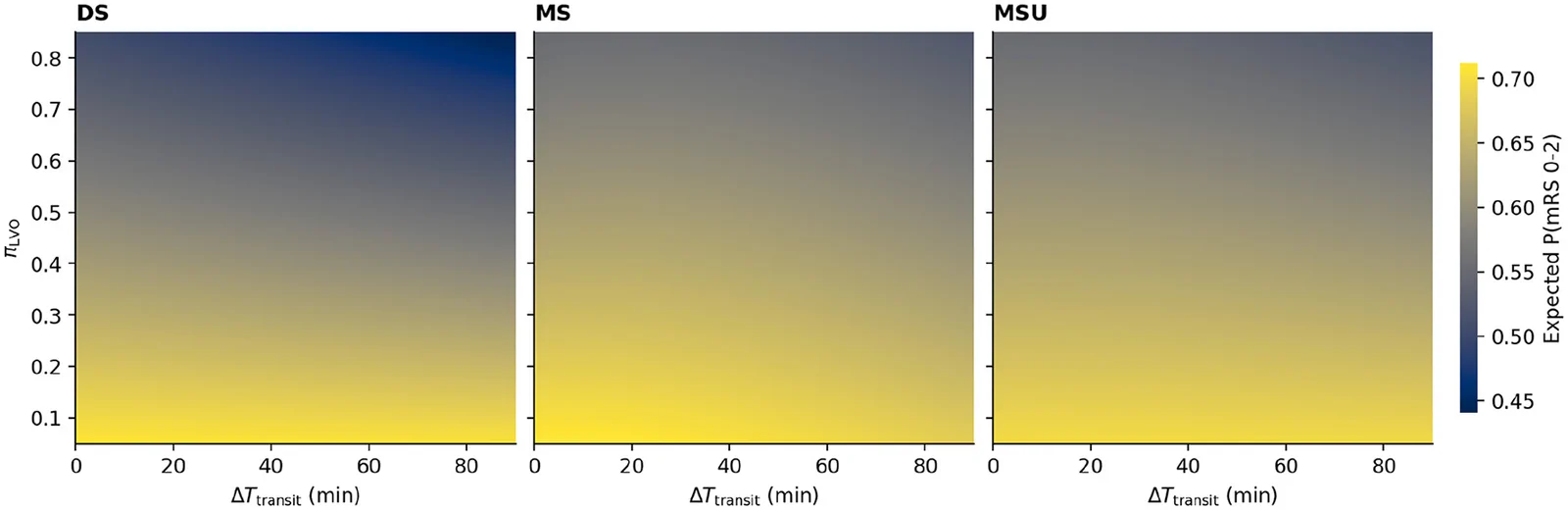
Expected 90-day favourable-outcome probability *E*[*p*_*a*_] under the tenecteplase calibration. **Left**: drip-and-ship. **Centre**: mothership. **Right**: mobile stroke unit.

Figure 5 displays the expected favourable-outcome probability *E*[*p*_*a*_] over the decision plane for each strategy under the tenecteplase calibration. The surfaces clarify the boundary partition: mothership dominates in three of the four quadrants, and the mobile-stroke-unit corridor is confined to the low-LVO/long-differential quadrant under tenecteplase calibration (29.0% of cells in that quadrant), expanding to 58.3% under alteplase calibration. The drip-and-ship surface remains uniformly inferior to mothership above π_LVO_≈0.3 at all transit differentials evaluated.

### 4.6 Posterior credible regions on the optimal strategy

**Figure 6:**
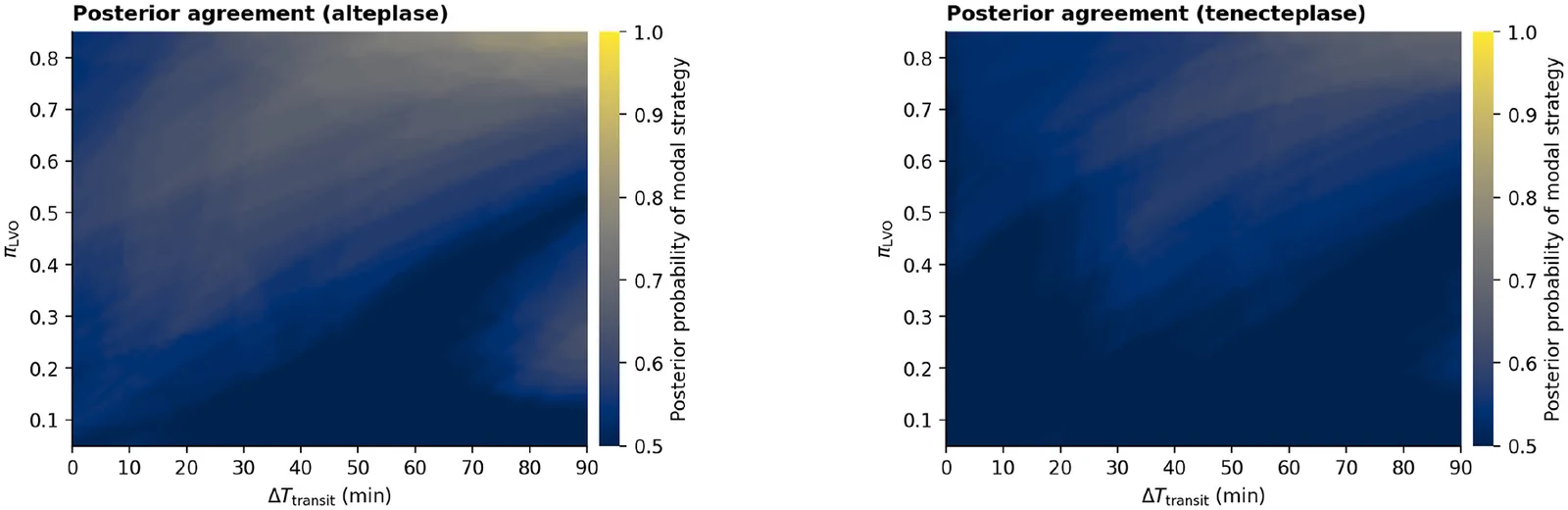
Agreement fraction on the modal optimal strategy under anchor-perturbed calibration-uncertainty resampling. **Left**: alteplase. **Right**: tenecteplase. Colour scale truncated at 0.5–1.0.

Calibration-uncertainty resampling (150 anchor-perturbed draws) produced the agreement maps in Figure 6. No grid cell achieved agreement ≥0.95 on its modal strategy under either pharmacological calibration; mean agreement exceeds 0.80 only along a narrow stripe at very high π_LVO_ and small Δ*T*_transit_. The maps reflect the propagated uncertainty in the published time-decay anchor probabilities at the perturbation standard errors specified in Section 3.10; the agreement fraction is not interpreted as a posterior credibility statement in the formal Bayesian sense.

### 4.7 Sobol global sensitivity

Variance-based decomposition under the Saltelli A/B/AB_*i*_ design (28, 29), with inputs sampled from independent uniform ranges (parameter-range sensitivity, not joint-posterior decomposition), attributes the dominant share of outcome variance to π_LVO_ in every strategy (Table 4). For the mothership branch $S_{\pi_{\text{LVO}}}^{\left(1\right)}=0.842$ and $S_{\pi_{\text{LVO}}}^{T}=0.844$, leaving very little residual variance for higher-order interactions in this strategy. Transit-time differential and symptom-to-call interval each contribute 0.06–0.08 to the first-order index, with similar total-order indices indicating weak interaction effects under the IVT/EVT-split workflow. The drip-and-ship and mobile-stroke-unit branches concentrate even more variance in π_LVO_ (first-order indices 0.927 and 0.932 respectively); the door-to-needle and door-to-puncture multipliers contribute negligibly to first-order variance (*S*^(1)^ < 0.003), reflecting that intra-hospital workflow times are tightly constrained relative to transport-time variability. Convergence diagnostics at *N*∈{256, 1024, 4096} for the mothership strategy show first-order index drift bounded by 0.001 between *N* = 1024 and *N* = 4096 (30), supporting *N* = 2048 as the production setting.

**Table 4:** Sobol first-order *S*^(1)^ and total-order *S*^*T*^ indices at *N* = 2, 048 for the tenecteplase calibration.

**Factor**	MS *S*^(1)^	MS *S*^*T*^	DS *S*^(1)^	MSU *S*^(1)^
π_LVO_	0.842 ± 0.047	0.844 ± 0.040	0.927 ± 0.049	0.932 ± 0.050
*T*_transit, PSC_	0.005 ± 0.005	0.007 ± 0.001	0.009 ± 0.006	0.002 ± 0.003
Δ*T*_transit_	0.077 ± 0.018	0.087 ± 0.006	0.024 ± 0.012	0.028 ± 0.013
*T*_call_	0.060 ± 0.016	0.069 ± 0.005	0.027 ± 0.011	0.012 ± 0.010
*T*_resp_	0.001 ± 0.003	0.001 ± 0.000	0.000 ± 0.002	0.000 ± 0.003
DTN_PSC_ mult.	0.000	0.000	0.001 ± 0.002	0.000
DTP_CSC_ mult.	0.002 ± 0.003	0.003 ± 0.000	0.002 ± 0.003	0.002 ± 0.003

Values are means ± bootstrap 95% confidence-interval half-widths. MS, mothership; DS, drip-and-ship; MSU, mobile stroke unit.

### 4.8 Stochastic dominance

**Figure 7:**
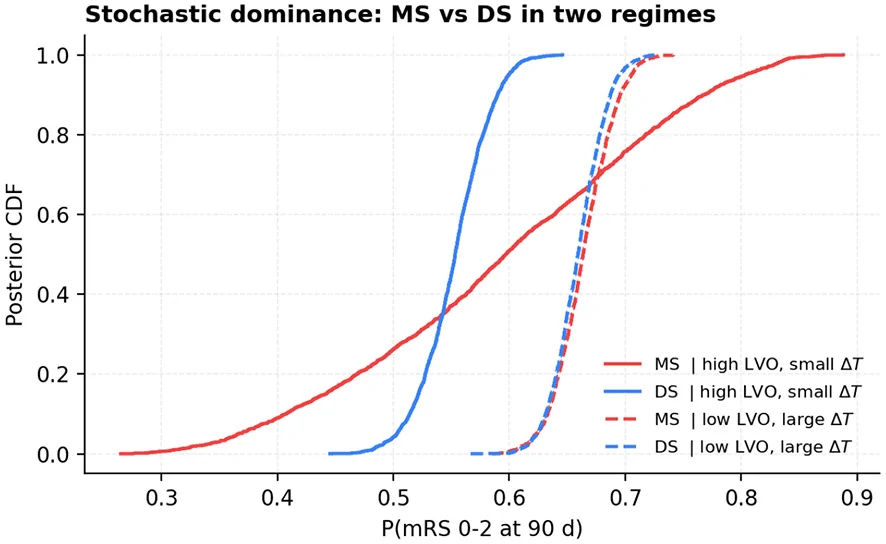
Posterior cumulative distribution functions of the 90-day favourable-outcome probability for mothership and drip-and-ship in two contrasting decision-plane regimes. Tenecteplase calibration; 2,000 anchor-perturbed Monte Carlo draws.

Posterior outcome samples (*n* = 2, 000 per strategy) at the high-LVO/short-differential regime (π_LVO_ = 0.65, Δ*T*_transit_ = 10 min) gave mean P(mRS 0–2) of 0.594 (mothership) versus 0.552 (drip-and-ship). Their cumulative distribution functions cross, so first-order stochastic dominance fails in both directions; second-order dominance also fails (sup difference 0.041). At the low-LVO/long-differential regime (π_LVO_ = 0.20, Δ*T*_transit_ = 70 min) the means are 0.664 (mothership) versus 0.659 (drip-and-ship); neither order of dominance can be asserted. The mobile-stroke-unit marginal benefit relative to mothership in the LVO subgroup (π_LVO_ = 0.70, Δ*T*_transit_ = 20 min) was −0.0072 under alteplase and −0.0053 under tenecteplase: at modest geographic separations the on-scene treatment delay before downstream EVT puncture is not offset by the onset-to-needle reduction (8, 9). Empirical cumulative distribution functions for the two regimes are shown in Figure 7.

### 4.9 Sensitivity to the LVO screening operating point

Post-screening LVO posteriors at three reference prevalences π_0_∈{0.10, 0.21, 0.40} are reported in Table 5. At baseline prevalence 0.21 a screen-positive RACE patient enters the decision plane near the lower edge of the mothership-favoured region under the alteplase calibration, while a screen-positive ACT-FAST patient enters well above the mothership/drip-and-ship boundary under either pharmacological calibration. The null overall mothership-versus-drip-and-ship effect reported by the RACECAT cluster-randomised trial (20) (90-day mRS common odds ratio 1.03) is consistent with the alteplase-calibration partition being approximately balanced once posterior uncertainty is propagated.

**Table 5:** Post-screening LVO posterior probabilities under the RACE ≥ 5 cutoff (27) and the ACT-FAST 3-step algorithm (10), at base prevalences π_0_.

			PPV at π_0_			π_**−**_**at** π_**0**_	
Scale	Sens.	Spec.	0.10	0.21	0.40	0.10	0.40
RACE ≥ 5	0.85	0.68	0.228	0.414	0.639	0.024	0.128
ACT-FAST	0.857	0.935	0.594	0.778	0.898	0.017	0.093

PPV is the post-screening LVO probability on a positive scale; π_−_ is the post-screening LVO probability on a negative scale.

### 4.10 External face-validity against landmark trials

**Figure 8:**
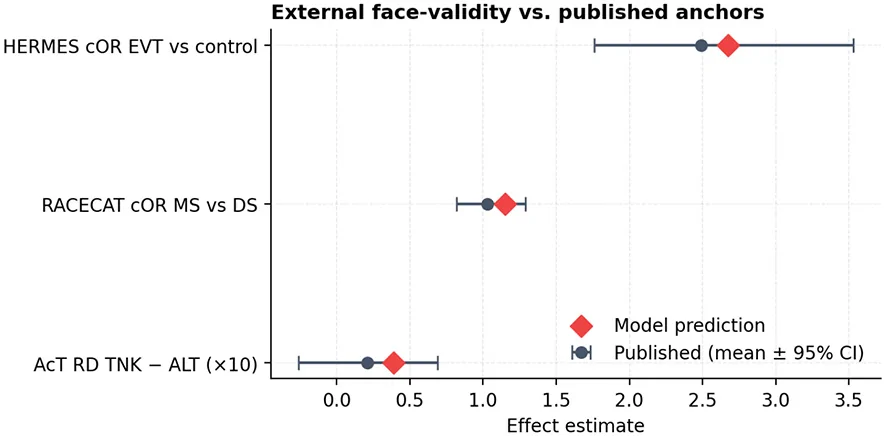
External face-validity of the conditional outcome model against three landmark trials. Markers: model point estimates. Bars: published 95% confidence intervals. HERMES: EVT vs control common odds ratio at 270-min onset-to-puncture. RACECAT: mothership vs drip-and-ship common odds ratio at π_LVO_ = 0.414, Δ*T*_transit_ = 30 min. AcT: tenecteplase vs. alteplase risk difference at 120-min onset-to-needle.

Three reference points were evaluated against the present model without re-fitting (Figure 8, Table 6). At the HERMES median onset-to-puncture window of 270 minutes the model returned an EVT-versus-control common odds ratio of 2.68, inside the published HERMES pooled common odds ratio of 2.49 (95% CI 1.76–3.53) (2). At the RACECAT operating point (π_LVO_ = 0.414, Δ*T*_transit_ = 30 minutes) the model returned a mothership-versus-drip-and-ship common odds ratio of 1.15, inside the RACECAT 90-day mRS common odds ratio of 1.03 (95% CI 0.82–1.29) (20). At the AcT 120-min reference window the model returned a tenecteplase-versus-alteplase risk difference of +0.039, inside the AcT 95% CI of −0.026 to +0.069 around the observed +0.021 (16).

**Table 6:** External face-validity values.

**Reference**	Endpoint	Model	Published (95% CI)
HERMES; (2)	EVT vs control cOR (270 min)	2.68	2.49 (1.76–3.53)
RACECAT; (20)	MS vs DS cOR (π = 0.41, Δ*T* = 30 min)	1.15	1.03 (0.82–1.29)
AcT; (16)	TNK vs ALT RD (120 min)	+0.039	+0.021 (−0.026, +0.069)

Model estimates are produced from the production calibration of Section 3.10; published values are reproduced from the cited landmark trials.

### 4.11 Sensitivity to the anchor-perturbation magnitude

The agreement maps of Section 4.6 use a per-anchor perturbation standard deviation σ_*g*_ = 0.06 on the EVT branch and σ_*h*_ = 0.04 on the IVT branches. To establish that the absence of high-agreement cells is not an artefact of the chosen σ, the resampling was repeated at σ_*g*_∈{0.03, 0.06, 0.10} with σ_*h*_ scaled in fixed ratio (Table 7). At the most optimistic setting (σ_*g*_ = 0.03) only 2.6% of grid cells reach agreement ≥0.80 on the modal strategy and zero cells reach agreement ≥0.95; at the production setting and the conservative setting these fractions collapse to zero. Mean agreement decreases monotonically with σ, from 0.626 to 0.502, confirming that the spatial extent of strategy ambiguity is robust to the perturbation magnitude over the plausible range.

**Table 7:** Anchor-perturbation sensitivity sweep.

σ_*g*_	σ_*h*_	Decisive ≥0.80	Decisive ≥0.95	Mean agreement
0.03	0.02	0.026	0.000	0.626
0.06	0.04	0.000	0.000	0.547
0.10	0.08	0.000	0.000	0.502

σ_*g*_ is the per-anchor perturbation standard deviation for the EVT branch; σ_*h*_ for the IVT branches. Decisive fractions are the share of grid cells whose modal-strategy agreement crosses 0.80 or 0.95.

## 5 Discussion

### 5.1 Principal findings

The simulation produced a partition of the (π_LVO_, Δ*T*_transit_) plane that is materially dependent on the assumed intravenous thrombolytic. Substituting tenecteplase for alteplase reallocated 14.1% of grid cells, with the change concentrated in the low-LVO/long-differential quadrant where the alteplase calibration favours the mobile stroke unit but the faster tenecteplase bolus reinstates mothership transport. Anchor-level uncertainty—perturbations of the published time-decay anchor probabilities at clinically defensible standard errors—was sufficient to render the optimal recommendation non-deterministic across the entire decision plane, with no cell achieving ≥0.95 posterior probability on its modal label, a finding that is robust to the perturbation magnitude across the sweep σ_*g*_∈{0.03, 0.06, 0.10}. Across strategies the prehospital LVO posterior accounted for the majority of outcome variance (Sobol *S*^(1)^ = 0.84–0.93); transit-time differential and symptom-to-call interval contributed the next-largest fractions, while the door-to-needle and door-to-puncture multipliers contributed negligibly, reflecting the tight constraint on intra-hospital workflow times relative to transport-time variability. Because the optimal label is non-deterministic across the entire plane, the boundaries reported here are best read as scenario-dependent expected-value contours rather than fixed thresholds: their position moves with the assumed transfer time, LVO prevalence, onset uncertainty and treatment-effect coefficients, and the anchor-perturbed agreement maps (Figure 6) quantify that movement directly rather than concealing it behind a single deterministic line.

### 5.2 Relation to prior conditional-probability frameworks

The framework extends the deterministic decision-curve constructions of (6) and the multi-strategy decision-analytic comparisons of (11, 32) in three respects. The treatment-effect priors are constructed by explicit Bayesian random-effects synthesis (25, 26), so the conditional-probability inputs inherit calibrated heterogeneity rather than being pinned to the point estimates of a single trial. Sensitivity is decomposed by a Saltelli A/B/AB_*i*_ design at *N*(*k*+2) = 18432 evaluations (28–30), replacing the one-at-a-time perturbations common in earlier work and yielding bootstrap-bounded indices with explicit convergence diagnostics. Preference orderings between strategies are established or ruled out by stochastic dominance (31) without committing to a single utility specification, in line with prior calls to avoid arbitrary scalarisation in stroke decision modelling.

### 5.3 Interpretation of the tenecteplase-era boundary shift

The redistribution of optimal cells toward drip-and-ship under the tenecteplase calibration is driven by the mechanism that distinguishes the two pharmacologies in the IVT branch of the outcome model. Tenecteplase, with its 14-fold higher fibrin specificity and single-bolus administration (15, 19), sustains a higher non-LVO good-outcome curve *h*_TNK_(*T*) than *h*_ALT_(*T*) at matched *T*_OTR_; the LVO curve *g*_EVT_(*T*) is shared between the two paradigms because both eventually pursue endovascular thrombectomy. Differential elevation of *h* favours strategies that deliver the bolus earliest—in this simulation, drip-and-ship at the primary stroke centre—whenever the geographic differential is large enough that the secondary transit penalty does not offset the IVT-arm gain. The Δ*T*_transit_≈25 min crossover implied by Figure 4 is in line with the AcT trial (16) and the seven-trial pooled analysis of (17): tenecteplase establishes its non-inferiority and modest superiority precisely in the regime where IVT decisions cannot be deferred until after a long transfer.

### 5.4 Implications for pre-hospital triage protocols

The variance decomposition makes the LVO posterior the single most consequential input. Even small improvements in scale calibration—whether of RACE (27) or ACT-FAST (10)—translate into outsized reductions in outcome variance, more than additional minutes shaved from any single time component, so investments in scale validation, paramedic training, and Bayesian fusion of multiple scales are favoured by the present analysis. The redistribution of the optimal partition under tenecteplase has its own protocol implication: bypass rules developed during the alteplase era (20) require re-derivation rather than parameter tuning when a network adopts tenecteplase as the standard IVT agent, because the boundary changes are not adequately captured by simple threshold updates. Finally, the mobile-stroke-unit benefit observed in BEST-MSU (8) and B_PROUD (9) is most clearly preserved at long secondary-transit differentials and high π_LVO_; in dense urban networks with short inter-hospital separations, the conditional-probability model finds the on-scene treatment delay difficult to recover under the present MSU parameterisation, the limits of which are stated in Section 5.6.

### 5.5 Clinical implications and decision framing

Translated into operational terms, the variance decomposition and the dominance results carry three practical messages. First, the single most consequential quantity for the routing decision is the prehospital LVO posterior, not any individual process-time component (Sobol first-order index 0.84–0.93 across strategies versus < 0.09 for every time component); the practical corollary is that the accuracy and the local calibration of the LVO screening scale dominate the decision, and that the transportability of scale performance across populations—which varies materially between the derivation cohorts of RACE (27) and ACT-FAST (10) and any given EMS system—is the principal threat to the external validity of a boundary map. Second, because neither first- nor second-order stochastic dominance could be established between mothership and drip-and-ship in the tested regimes, the strategy ranking is fragile rather than robust: it depends on the decision-maker's attitude to risk and cannot be settled on outcome distributions alone. If expected favourable-outcome probability is adopted as the sole criterion, the choice reduces to a marginal comparison of means, which the present model resolves only weakly; if instead a risk-averse posture is taken—weighting the avoidance of severe disability more heavily than the mean—the second-order dominance test provides the appropriate formal instrument, and its failure to separate the strategies indicates that guidelines should make the assumed risk stance explicit rather than presenting a single strategy as unconditionally superior. Third, the tenecteplase-era reallocation is concentrated in the low-LVO, long-differential region, so the operational impact of adopting tenecteplase is felt mainly by patients screening at lower LVO probability who face long secondary transfers, not by high-probability LVO patients near a comprehensive centre.

Box 1 **Clinical implications at a glance.**

- High posterior LVO probability (for example a positive result on a high-specificity scale) favours mothership transport across almost the entire range of transit-time differentials under both pharmacologies.
- Low-to-moderate LVO probability with a comprehensive centre substantially farther than the nearest primary centre is the only region in which drip-and-ship or the mobile stroke unit becomes competitive; adopting tenecteplase shifts part of this region from the mobile stroke unit toward mothership or drip-and-ship.
- Intra-hospital door-to-needle and door-to-puncture times matter far less to the optimal choice than LVO screening accuracy and the inter-facility transit differential; investment in scale calibration and paramedic training is favoured over marginal intra-hospital time savings.
- No coordinate of the decision plane reached 95% posterior agreement on a single strategy; the maps are decision-support contours to be calibrated locally, not fixed triage rules.

### 5.6 Limitations

Several limitations constrain the strength of the applied claims. The time-decay coefficients are fitted from five anchor points (180, 270, 360, 450, 540 min) on the EVT branch and seven anchor points (60, 90, 180, 270, 360, 450, 540 min) on the IVT branches, traceable to (1, 2, 4, 5); with three coefficients per parabolic logit and five or seven anchors the fit retains two or four residual degrees of freedom respectively, but extrapolation outside the anchor range remains unsupported. The mobile-stroke-unit branch is now modelled with a separate downstream onset-to-puncture time for LVO patients (Section 4.10); the residual MSU underperformance at modest geographic separations (Section 4.8) is therefore a property of the anchored time-decay curves under realistic comprehensive-stroke-centre door-to-puncture parameters, not of the earlier coarse parameterisation, but should still be read against the predictive interval of the BEST-MSU/B_PROUD pair (0.90–3.20) rather than as a single point estimate. Intra-hospital handoff and door-in-door-out times are represented by point values calibrated to published averages rather than network-specific measurements; the Sobol indices report sensitivity to these parameters, but do not substitute for empirical measurement in any specific network. The Sobol decomposition itself samples inputs from independent uniform ranges and is therefore a parameter-range sensitivity analysis rather than a fully specified joint-posterior decomposition; correlations between input parameters that hold in any specific network are not represented. The simulation also assumes a single LVO scale defines π_LVO_, whereas in practice the posterior would reflect a fusion of multiple scales, dispatcher information, and possibly imaging triage. Capacity constraints and patient-flow queueing are not represented: in saturated comprehensive-stroke-centre networks the optimal individual recommendation may diverge from the network-optimal allocation. Lastly, the random-effects synthesis treats the published treatment-effect estimates as exchangeable; trial-level features such as imaging selection (4, 5) and time window are not modelled as moderators.

Several of these constraints bear directly on real-world translation. The treatment-effect anchors are drawn from randomised trials and individual-patient meta-analyses whose enrolled populations, workflow discipline and geography may differ substantially from unselected prehospital EMS populations; trial-derived coefficients therefore represent an efficacy ceiling that may not transfer cleanly to heterogeneous field conditions, and local calibration would be required before any operational use. Because the Sobol inputs are sampled from independent uniform supports, the reported indices describe sensitivity to plausible parameter ranges in isolation; in a specific stroke network the same inputs are correlated—transit differential with LVO prevalence through geography, and door-to-needle with door-to-puncture through shared staffing—so the variance shares should be read as network-agnostic screening quantities rather than as the exact apportionment of uncertainty for any one system's policy-making. The mobile-stroke-unit branch is modelled with a static onset-to-puncture penalty and does not represent dynamic availability constraints: a unit already committed to another dispatch, or delayed by traffic or adverse weather, effectively enlarges the transit differential in real time and can move the optimal strategy away from the value implied by the static map. In the early time window below approximately 120 minutes, where the tenecteplase bolus advantage is most pronounced, the three-parameter quadratic-logit decay is constrained by only the earliest anchor probabilities, so structural misspecification of the curve shape in this region cannot be excluded despite the reassuring external face-validity checks; and the log-normal process-time family, although consistent with the cited registries, was not tested against alternative positive-support families such as the gamma or Weibull. Most consequentially, the framework has not been externally validated against prospective regional stroke-system outcomes; this is a central rather than peripheral limitation, and the conclusions are framed accordingly as decision-analytic and hypothesis-generating rather than operationally confirmatory.

### 5.7 Future directions

A natural extension is the inclusion of clot-length and imaging-derived predictors as moderators of the intravenous–endovascular interaction (18), refining the LVO branch of the outcome decomposition for patients with proximal occlusions exceeding 10 mm. Telerobotic endovascular therapy could enter the framework as a fourth strategy, with operator-side latency and per-procedure success probability handled within the same Bayesian-prior pipeline. The conditional-probability optimum can also be composed with bed-availability and angiosuite-utilisation queueing models, lifting the present per-patient recommendation to network-level allocation under capacity constraints.

## 6 Conclusion

A Bayesian conditional-probability simulation framework was developed to evaluate drip-and-ship, mothership, and mobile-stroke-unit pre-hospital transport strategies under the tenecteplase-era reperfusion calibration. Random-effects synthesis of treatment-effect priors, deterministic decision-boundary mapping, anchor-perturbed posterior credible regions, Saltelli-design Sobol global sensitivity, and Davidson–Duclos stochastic dominance tests were composed into a single reproducible pipeline. Substituting tenecteplase for alteplase reallocated 14.1% of the joint (π_LVO_, Δ*T*_transit_) decision plane, with the change concentrated in the low-LVO/long-differential quadrant where the faster tenecteplase bolus reinstates mothership transport in cells previously assigned to the mobile stroke unit; the prehospital LVO posterior accounted for the dominant share of outcome variance across all strategies (Sobol *S*^(1)^ = 0.84–0.93); and the mobile-stroke-unit advantage in the LVO subgroup was attenuated under the tenecteplase calibration at modest geographic separations. Anchor-level uncertainty alone was sufficient to render no cell of the decision plane deterministically optimal at the 95% posterior level, indicating that future calibration efforts should target the time-decay anchors of the endovascular branch as a priority over additional refinement of the time-component priors . Consistent with the modelling nature of the study, these results are offered as a structured and reproducible means of evaluating routing strategies under uncertainty rather than as definitive guidance on what emergency medical systems should do in practice; prospective validation in real-world regional stroke networks is warranted before the framework informs operational triage protocols. The complete pipeline is openly released at https://github.com/tugbacalisir/frontiers under the MIT licence.

## Data Availability

The datasets presented in this study can be found in online repositories. The names of the repository/repositories and accession number(s) can be found in the article/supplementary material.
